# Designing a Long Acting Erythropoietin by Fusing Three Carboxyl-Terminal Peptides of Human Chorionic Gonadotropin **β** Subunit to the *N*-Terminal and *C*-Terminal Coding Sequence

**DOI:** 10.1155/2011/275063

**Published:** 2011-08-21

**Authors:** Fuad Fares, Avri Havron, Eyal Fima

**Affiliations:** ^1^Department of Human Biology, Faculty of Natural Sciences, University of Haifa and Department of Molecular Genetics, Carmel Medical Center, Mount Carmel, 31905 Haifa, Israel; ^2^ModigeneTech, Weizmann Science Park, 74140 Nes-Ziona, Israel; ^3^PROLOR Biotech, Weizmann Science Park, 74140 Nes-Ziona, Israel

## Abstract

A new analog of EPO was designed by fusing one and two CTPs to the *N*-terminal and *C*-terminal ends of EPO (EPO-(CTP)_3_), respectively. This analog was expressed and secreted efficiently in CHO cells. The *in vitro* test shows that the activity of EPO-(CTP)_3_ in TFI-1 cell proliferation assay is similar to that of EPO-WT and commercial rHEPO. However, *in vivo* studies indicated that treatment once a week with EPO-(CTP)_3_ (15 *μ*g/kg) dramatically increased (~8 folds) haematocrit as it was compared to rHuEPO. Moreover, it was found that EPO-(CTP)_3_ is more effective than rHuEPO and Aranesp in increasing reticulocyte number in mice blood. The detected circulatory half-lives of rHuEPO, Aranesp, and EPO-(CTP)_3_ following IV injection of 20 IU were 4.4, 10.8, and 13.1 h, respectively. These data established the rational for using this chimera as a long-acting EPO analog in clinics. The therapeutic efficacy of EPO-CTP analog needs to be established in higher animals and in human clinical trials.

## 1. Introduction

Erythropoietin (EPO) is a 34-kDa glycoprotein hormone produced primarily by cells of the per tubular capillary endothelium of the kidney and regulates red blood cell production through stimulation of erythropoiesis [1, 2]. EPO synthesis in the kidney is increased following reduction in tissue oxygenation, it binds to specific receptors on red blood cell precursors in the bone marrow leading to proliferation, differentiation, and to an increase in haematocrit [3]. Biological responses associated with EPO include activation of intracellular signaling molecules such as transcription factors like signal transducer and activator of transcription (STAT) proteins leading to cellular growth and differentiation. EPO receptor belongs to a family of homodimerization receptors where dimerization of the receptor is required to trigger the biological responses associated with EPO [4–7]. Anemia in patients with chronic kidney disease is due to a number of factors, the most common of which is abnormally low erythropoietin levels. Anemia of EPO deficiency is recognized in advanced renal failure but not in early renal disease. Deficiency in EPO production results in anemia in humans and in animal models. EPO is heavily glycosylated with one *O*-linked and three *N*-linked oligosaccharide chains. It was found that *O*-linked oligosaccharide chain has no effect on secretion, receptor binding affinity, and *in vitro* or *in vivo* bioactivity. On the other hand, *N*-linked oligosaccharide- chains have no role in *in vitro* activity, but it is critical for *in vivo* bioactivity [8]. 

The gene encoding human erythropoietin was cloned in 1985 leading to the production of recombinant human EPO (rHuEPO) [9, 10]. rHuEPO was used successfully in treating anemia associated with chronic kidney disease. It has also been approved for the treatment of anemia associated with cancer, HIV infection, and in the surgical setting in order to reduce blood transfusions [11–13]. One major issue regarding the clinical use of EPO is its relatively short half-life *in vivo* due to its rapid clearance (~5 hours) from the circulation when it is injected intravenously [14]. Thus, the clinical therapeutic protocols of available stimulating agents used in the treatment of patients require frequent injections of EPO. The recommended therapy with rHuEPO is 2-3 times per week by subcutaneous or intravenous injections. Therefore, it can be anticipated that enhancing the *in vivo* half-life of EPO would reduce the number of injections per week. Previous studies indicated that there is a direct relationship between the sialic acid-containing carbohydrate content of the molecule and its serum half-life and *in vivo *bioactivity [15–17]. It was shown that fusing the carboxyl-terminal peptide (CTP) of hCG*β* subunit that associated with four sites of *O*-linked oligosaccharide chains to the *C*-terminal of FSH, TSH, GH, and EPO cDNA did not affect secretion, receptor binding affinity, and *in vitro *bioactivity. On the other hand, the addition of *O*-linked oligosaccharides to the backbone of the protein significantly increased the half-life and longevity *in vivo* [18–22]. We hypothesis that the addition of 12 *O*-linked oligosaccharide chains to the backbone of EPO will dramatically increase the longevity of EPO. Therefore, in the present study, three carboxyl-terminal peptides (CTP) of hCG*β* subunit that each contains four *O*-linked oligosaccharide recognition sites was fused to *N*-terminal (one) and to the *C*-terminal (two) of human EPO coding sequence, respectively. Our results indicate that ligation of three CTPs to the coding sequence of EPO dramatically increased both *in vivo* potency and half-life in the circulation.

## 2. Materials and Methods

### 2.1. Materials

Enzymes used in the construction of DNA vectors and constructs were purchased from New England BioLabs (Beverly, Mass, USA). Cell culture media and reagents were obtained from Biological Industries (Beit Haemek, Israel). Rabbit antisera against EPO were purchased from Fitzgerald (Concord, Mass, USA). The eukaryotic expression vector (pCI-DHFR, Dihydrofolate reductase) into which the cDNA encoding for the corresponding hEPO variants were inserted was purchased from Promega, (San Luis Obispo, Calif, USA). Commercial human recombinant EPO (Eprex) was purchased from Janssen-Cilag (North Ryde, NSW, Australia).

### 2.2. Crystallography

The interaction between EPO and its receptor was crystallized as described previously [23] by the Department of Structural Biology, Weizmann Institute of Science, Rehovot, Israel.

### 2.3. Construction of Chimeric Genes and Expression Vectors

**Figure 1:**
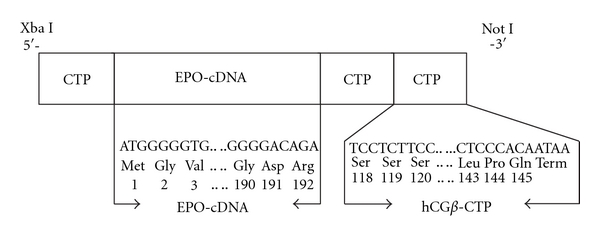
Construction of EPO-(CTP)_3_ chimeric gene. The chimeric gene contains the cDNA of human erythropoietin and three hCG*β* carboxyl-terminal peptides that were ligated to the *N*-terminal (one) and to *C*-terminal (two) coding sequences.

A cassette gene containing the CTP of hCG*β* was fused in tandem to the coding sequence of EPO at the *N*-terminal (one CTP) and to the *C*-terminal end (two CTPs) (Figure 1). DNA fragment containing sequences of hEPO-cDNA and coding sequence of CTP were synthesized by GeneArt (Regensburg, Germany). The DNA fragments contain the recognition sites of the restriction enzymes; *Xba I* (in the *N-terminal*) and *Not I* (in the *C*-terminal). Fragment containing hEPO and CTP sequences was completely sequenced to ensure that no errors were introduced during synthesis and ligated into the *XbaI*—*Not I* sites at the cloning site of the eukaryotic expression vector, pCI-DHFR. Similarly, cDNA of human EPO (EPO-WT) was constructed into pCI-DHFR vector. 

### 2.4. Cell Culture and DNA Transfection

Chinese hamster ovary (CHO)-DG44 cells, which are DHFR negative, were used. Cells were cultured in MEM-*α* medium (Gibco BRL, USA) supplemented with penicillin (100 U/mL), streptomycin (100 mg/mL), L-glutamine (2 mM), and 10% heat-inactivated fetal bovine serum at 37°C in humidified incubator containing 5% CO_2_. These cells were transfected with 2 *μ*g DNA of plasmid by using FuGENE6 (Roche, Mannheim, Germany) according to manufacturer protocol.

Cells were selected for insertion of the plasmid DNA by growth in culture medium of CD DG44 without hypoxanthine and thymidine (HT) (Gibco BRL, USA) supplemented with 8 mM L-Glutamine (Biological Industries, Beit Haimic, Israel) and 18 mL/L of 10% Pluronic F-68 solution (Gibco BRL, USA).

### 2.5. Western Blotting

Samples of condition medium which were collected from stable clones were electrophorised on denaturing 15% SDS-polyacrylamide gels as described before [24]. Gels were allowed to equilibrate for 10 min in 25 mM Tris and 192 mM glycine in 20% (vol/vol) methanol. Proteins were transferred to a 0.2 *μ*m pore size nitrocellulose membrane (Sigma, Saint Louis, Mo, USA) at 250 mA for 3 h using a Mini Trans-Blot electrophoresis cell (Biorad Laboratories, Richmond, CA) according to the method described in the manual accompanying the unit. The nitrocellulose membrane was incubated in 5% nonfat dry milk for 2 h at room temperature. The membrane was incubated with EPO antiserum (1 : 1000 titers) for overnight at 4°C followed by three consecutive washes in PBS containing 0.1% Tween (10 min/wash). Then, the membrane was incubated with secondary antibody conjugated to Horse Radish Peroxidase (HRP) (Zymed, San Francisco, CA) for 2 h at room temperature followed by three washes. Finally, the nitrocellulose paper was reacted with enhanced chemiluminescent substrate (ECL) (Pierce, Rockford, Ill, USA) for 5 min, dried with Whatman sheet and exposed to X-ray film.

### 2.6. *In Vitro* Bioactivity

Bioactivity of EPO variants was assayed by testing the proliferation dependence of the human erythroleukemic cell line TF-1 (Kitamura) (DSMZ) in the presence of EPO and EPO variants [25]. Cultures were routinely grown at 37°C, 5% CO_2_ for 72 hrs in RPMI 1640 medium supplemented with 10% fetal bovine serum (FBS),10 mM Hepes, 1 mM sodium pyruvate, 2.5 g/L glucose, 2 mM glutamine, and 2 ng/mL rhGM-CSF. Before transferring the cells to 96-well plates, the TF-1 cells were washed three times with cold PBS and suspended in the assay medium (1640 medium supplemented with 10% fetal bovine serum (FBS) but without addition of rHGM-CSF) at a density of 200,000 cells/mL. The assay was performed in 96-well plates containing 50 *μ*L of cell suspension per well. 50 *μ*L of assay medium containing 3 IU of EPO variants were then added to the wells of 96-well plates for 72 hrs. Cell viability was measured using MTT reagent kit (Cell Biolabs, San Diego, CA) according to manufacturer procedures.

### 2.7. Animals

Male ICR mice were obtained from Charles River Laboratories, Jerusalem, Israel, and housed in air-conditioned quarters with a 12 h light/dark schedule. Standard food and water were available *ad libitum*. Institute ethical committee approved the *in vivo* protocols. Animals were treated with EPO variants as specified.

### 2.8. *In Vivo* Bioassay 

Groups of 7 male ICR mice (7-week-old males) were used. EPO-WT, EPO-(CTP)_3_, or commercial rHuEPO were injected to anesthetized animals as described in Table 1. 

The animals were weighed, and each received an identical amount (5 or 15 *μ*g/kg) of EPO variants by *IV* injections (0.2 mL/animal). The frequency of treatment was either thrice weekly (days 1, 3, and 5) or once weekly. The level of haematocrit was determined three times a week and the experiment was stopped after three weeks. Haematocrit was determined using blood samples obtained by filling two heparinized microhematocrit tubes from the inferior caval vein under anesthesia. In addition, reticulocyte counts were conducted since these cells are present in blood for *∼*48 hours before developing into mature red blood cells. Reticulocytes represent an appropriate evaluation for the acute experimental system employed. Blood was obtained from each pup by cardiac puncture and placed in EDTA coated tubes. This was then mixed with brilliant cresyl blue and incubated for 20 min at 37°C. The blood and stain were then smeared onto a glass slide and the number of reticulocytes assessed using a × 100 oil objective lens.

### 2.9. Metabolic Clearance Rate

The metabolic clearance of EPO-WT, Aranesp, and EPO-(CTP)_3_ was determined after *IV* injection of 20 IU/animal into male ICR mice. At selected intervals after injection, blood samples were collected and EPO immunoreactivity was determined by RIA.

### 2.10. Statistical Analysis

Data were expressed as the mean ± SEM. Statistical analysis of the data were performed using Student's *t*-test and 1-way multivariate analysis of variance (ANOVA1) to calculate *P* value. *P* values < 0.05 were considered statistically significant.

## 3. Results and Discussion

**Figure 2:**
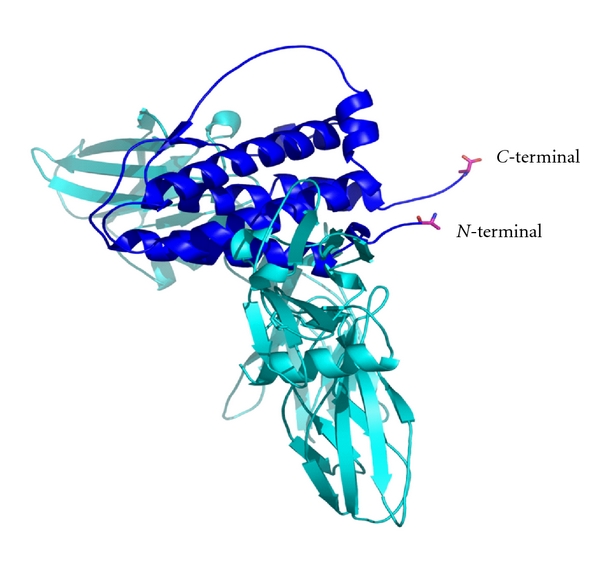
Model of erythropoietin binding to its receptor. The diagram showing the complex of EPO bound to the receptor. Note that the *N*-terminal and *C*-terminal of EPO are free and not involved in the binding site.

Chrystallographic studies indicated that the *N*-terminal and *C*-terminal of EPO are not involved in the binding of the hormone to the receptor (Figure 2). 

**Figure 3:**
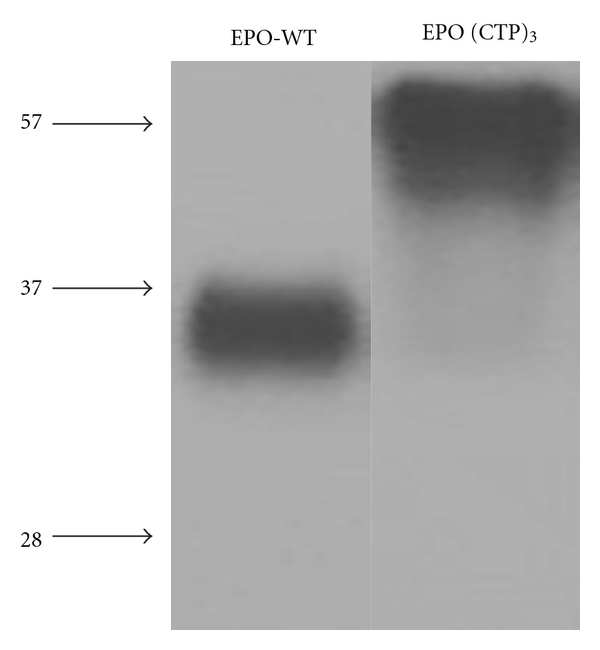
Expression of EPO-WT and EPO-(CTP)_3_ from transfected CHO cells. Conditioned media from transfected cells were prepared for SDS/PAGE and proteins were detected by Western blot as described under “Materials and Methods.”

Therefore, we hypothesized that ligation of CTP to the *N*-terminal and to the *C*-terminal of EPO will not affect receptor binding affinity and thus bioactivity. Therefore, three CTPs (one in the *N*-terminal and two in the *C*-terminal) were ligated to the coding sequence of EPO. The cDNA of human EPO-WT and EPO-(CTP)_3_ was inserted into the eukaryotic expression vector, pCI-DHFR, and transfected into CHO cells. Stable clone expressing human EPO-WT or EPO-(CTP)_3_ was selected. Secretion of EPO was assessed by Western blot analysis under denaturing conditions using human EPO-specific antiserum. The EPO-WT migrated faster than EPO-(CTP)_3_ (Figure 3). 

EPO-(CTP)_3_ exhibited high molecular weight (*∼*57 kDa) comparing to EPO-WT (*∼*36 kDa) due to the addition of 84 amino acids and the *O*-linked oligosaccharides linked to CTP. These data may indicate that the *O*-linked glycosylation recognition site of the *C*-terminal region is preserved even though the sequence is fused to different proteins. Levels of EPO-WT and EPO-(CTP)_3_ were quantities in condition medium by using a monoclonal antibody-based RIA. 

**Figure 4:**
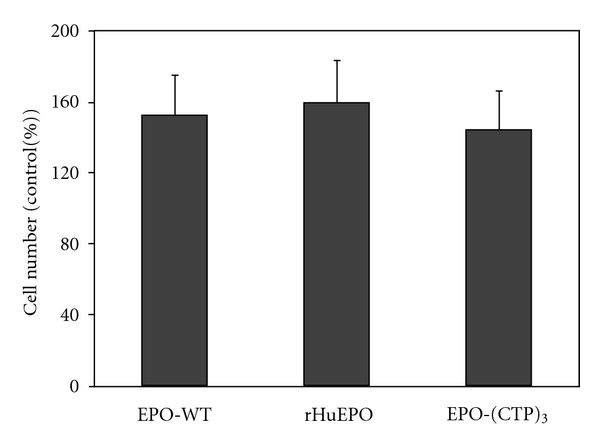
*In vitro *biological activity of recombinant hEPO derivatives. Bioactivity of EPO variants was tested by measuring the proliferation dependence of human erythroleukemicTF-1 cells in the absence or presence of 3 IU of EPO variants. Cell proliferation was measured using MTT reagent kit.

The *in vitro* biological activity of EPO analogs was demonstrated by measuring their ability to stimulate the proliferation of TF-1 cells as described under “Materials and Methods.” The activity of EPO-(CTP)_3_ in TF-1 cell proliferation assay was similar to that of EPO wild-type (prepared by Modigene Tech) and commercial rHuEPO (Figure 4).

**Figure 5:**
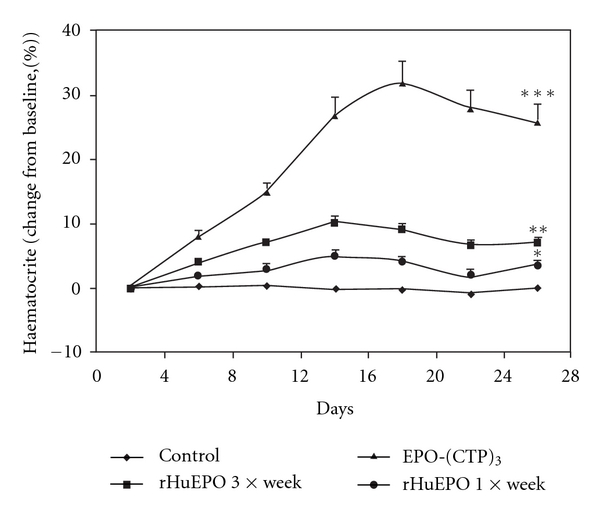
*In vivo* bioactivity of recombinant HuEPO derivatives. ICR mice (*n* = 7/group) received a single *IV* injection/week (15 mg/kg) for three weeks of EPO-WT, rHuEPO, or EPO-(CTP)_3_. In addition, mice were treated with 5 *μ*g/kg of rHuEPO three times a week for 3 weeks. Control animals were injected *IV* with saline. Blood samples were collected three times a week and haematocrit levels were detected. Each point represents the group average of haematocrit (%) ± SE. *P < 0.0.5, **P < 0.01, and ***P < 0.001.

For further pharmacological evaluation of EPO-(CTP)_3_, comparative pharmacodynamic studies of EPO-(CTP)_3_ and commercial rHuEPO were performed in male ICR mice (*n* = 7/group) using different frequencies and dose range as described in Table 1. The *in vivo* efficacy was obtained by measuring the mean values of haematocrit percentage in the blood. The results indicated that EPO-(CTP)_3_ is significantly (*P* < 0.001) more efficient than rHuEPO when administered *IV* once a week with a dose of 15 *μ*g/kg (Figure 5). EPO-(CTP)_3_ can successfully increase the haematocrit when administered once a week with a dose of 15 *μ*g/kg (Figure 5). Once weekly dosing with the same concentration of commercial rHuEPO or EPO-WT was significantly (*P* < 0.001) less efficient than once weekly dosing of EPO-(CTP)_3_. An interesting observation from the present study was the ability of a single injection once a week of EPO-CTP (15 *μ*g/kg) to increase dramatically (*∼*8 folds) the levels of haematocrit. Whereas administration of the same total dose of rHuEPO administered three times a week as 5 *μ*g/kg per injection resulted in significantly (*P* < 0.001) lower effect (Figure 5).

Previously, we have shown that single injection once a week of EPO-CTP, an EPO that contains one CTP at the carboxyl-terminal end, (15 *μ*g/kg) increased the level of haematocrit, whereas the same effect was achieved by administration of the same total dose of rHuEPO administered three times a week as 5 *μ*g/kg per injection [21]. These results indicated the importance of sustained blood levels, rather than total dose of EPO. These findings are consistent with the hypothesis that the ability of a single injection of EPO-CTP to increase haematocrit results from its increased stability in the circulation.

**Figure 6:**
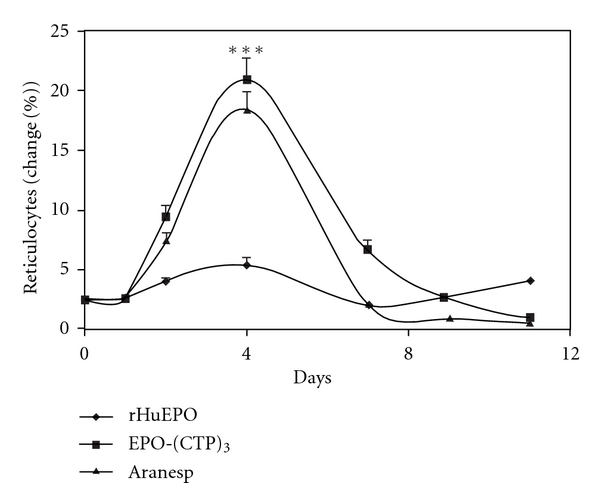
The effect of a single *IV* injection of EPO variants on reticulocyte counts in mice. ICR mice (*n* = 7/group) received a single *IV* injection/week for three weeks of rHuEPO, Aranesp, or EPO-(CTP)_3_ (15 mg/kg). Blood samples were collected after 72 h and reticulocytes were counted. Each point represents the group average of reticulocyte (%) ± SE. ***P < 0.001.

**Figure 7:**
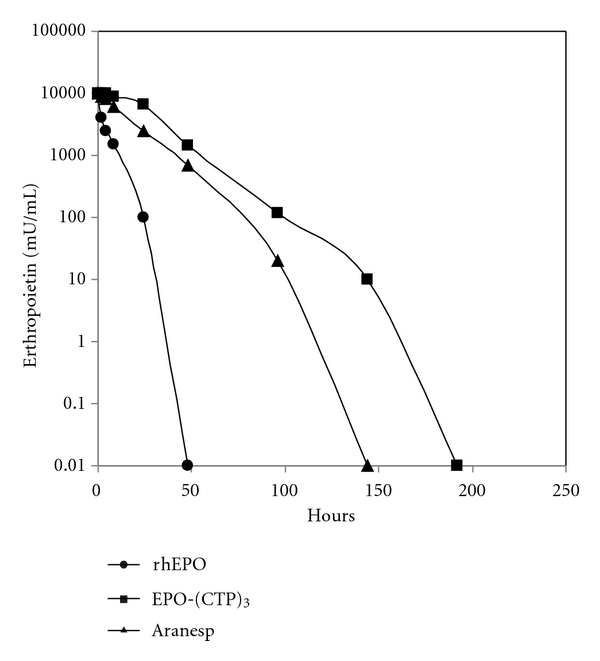
*In vivo* half-life of EPO variants. Mice were injected *IV* with 20 IU of rHuEPO, Aranesp and EPO-(CTP)_3_ and blood samples were drawn at the indicated times. Serum levels of EPO were determined by RIA. Mean ± SE of 5 determinations. Basal EPO levels before treatment were unmeasured.

Effect of EPO-WT, (EPO-CTP)_3_, and Aranesp in reticulocyte counts is shown in Figure 6. The results indicated that a single *IV* injection of 15 *μ*g/kg (EPO-CTP)_3_ dramatically increased reticulocyte number compared to rHuEPO and to Aranesp. The increased biopotency of the chimera may reflect a change in their *in vivo* longevity. Therefore, the circulatory half-lives of the hormones were determined. EPO-WT, Aranesp, or EPO-(CTP)_3_ was injected* IV* into immature male mice and RIA monitored the plasma half-lives. The results indicated that EPO-(CTP)_3_ has the highest half-life in circulation (Figure 7).

The estimated half-lives of EPO-WT, Aranesp, and EPO-(CTP)_3_ are 4.4, 10.8, and 13.1 hours, respectively (Table 2). These data suggest that the mechanism of EPO clearance is affected by the presence of CTP. Estimation of area under the curve (AUC) and the maximal plasma concentration (Cmax) of EPO-(CTP)_3_ are higher than that of rHuEPO and Aranesp. However, the maximal concentration reached in plasma (Tmax) is similar (Table 2). 

Previous studies indicated that the CTP sequence can be shuttled into different proteins and still be an acceptor for the *O*-linked oligosaccharides [18–20]. It was postulated that the *O*-linked oligosaccharides add flexibility, hydrophilicity, and stability to the protein [26]. This may explain the disinterference of CTP on the protein conformation and, thus, on receptor binding and bioactivity *in vitro*. On the other hand, it was suggested that the *O*-linked oligosaccharides play an important role in preventing plasma clearance and thus increasing the half-life of the protein in the circulation [18, 21, 22]. These roles have been postulated since the *O*-linked oligosaccharides are ended with sialic acid, which is negatively charged. It is known that negatively charged forms of the hormones are less cleared through the glomerular filtration [27]. Thus, addition of 12 *O*-linked oligosaccharide chains to the backbone of EPO significantly decreased renal clearance; the kidney is the main site of clearance for glycoprotein hormones and, thus, prolonged its half-life in the circulation. 

Other studies described long acting hyperglycosylated EPO analog that prepared by addition of *N*-linked oligosaccharides to the backbone of the protein. In order to add *N*-linked oligosaccharide chains, the DNA sequence of the cloned human EPO gene was modified by site-directed mutagenesis [28]. This analog was 3-fold longer serum half-life and created *in vivo* potency comparing to human recombinant EPO-WT. However, its relative affinity for the EPO receptor was *∼*4-fold lower than that of rHuEPO. Moreover, changing 5 amino acids in the backbone of the protein may increase the immunogenicity of the new derivative. 

Addition of CTP to the coding sequence of hormones FSH, TSH, and GH, do not affect secretion, receptor binding affinity, or bioactivity *in vitro* [18–22]. On the other hand, it was found that ligation of CTP to the coding sequence significantly increases the *in vivo* potency and half-life of the hormone. Moreover, it was found that hormone bearing CTP is safe for use in human and not immunogenic [29–31]. 

The present study describes a novel long-acting recombinant erythropoietin agonist designed by fusion of three CTP sequences to the coding sequence of EPO. This did not interfere with secretion or *in vitro* bioactivity. In contrast, addition of CTP sequences significantly increased the *in vivo* potency and half-life of EPO. These data establish a rationale for using this chimera as a long-acting EPO analog. However, the immunogenicity of this analog should be tested. Human erythropoietin has a wide clinical use in the treatment of anemia associated with a renal failure, HIV, and chemotherapy [32–36]. The therapeutic efficacy of this analog needs to be establishing in higher animals and in human clinical trials.

## Figures and Tables

**Table 1:** Comparative 3 week induction of haematocrit by EPO-(CTP)_3_ and rHuEPO.

Group number	Mice/group	Treatment		Regimen: IV
		Compound	Dose *μ*g/kg	
1	*n* = 7	Vehicle (control)	0	One dose per week
2		rHuEPO	15 *μ*g/kg	
3		EPO-(CTP)_3_	15 *μ*g/kg	
4		Commercial rHuEPO	5	3 doses per week

**Table 2:** Mean pharmacokinetic parameters following *IV* administration of a single dose (20 *μ*g/kg) of rHuEPO, EPO-(CTP)_3_, and Aranesp in male ICR mice. Parameters were generated for individual rats and the mean data are presented here.

Parameters	rHuEPO	EPO-(CTP)_3_	Aranesp
AUC (hr*μg/L)	31739	306072	178661
Cmax (*μ*g/L)	10766	16466	13266
Tmax (hr)	0.25	0.25	0.25
T1/2 (*α*) (hr)	4.4	13.11	10.84

## Abbreviations

EPO: Erythropoietin
RHuEPO: Recombinant human erythropoietin
WT: Wild-type
Hcg: Human Chorionic Gonadotropin
FSH: Follitropin
TSH: Thyrotropin
CTP: Carboxyl-terminal peptide
CHO: Chinese hamster ovary cells
PCR: Polymerase chain reaction
WT: Wild-type.
